# Reprint of “Investigating ensemble perception of emotions in autistic and typical children and adolescents”^☆^

**DOI:** 10.1016/j.dcn.2018.02.003

**Published:** 2018-02-21

**Authors:** Themelis Karaminis, Louise Neil, Catherine Manning, Marco Turi, Chiara Fiorentini, David Burr, Elizabeth Pellicano

**Affiliations:** a Centre for Research in Autism and Education (CRAE), UCL Institute of Education, University College London, UK; b Department of Psychology, Plymouth University, Plymouth, UK; c Department of Experimental Psychology, University of Oxford, Oxford, UK; d Department of Psychology, University of Florence, Florence, Italy; e Fondazione Stella Maris Mediterraneo, Chiaromonte, Potenza, Italy; f School of Psychology, University of Geneva, Switzerland; g School of Psychology, University of Western Australia, Perth, Australia

**Keywords:** Ensemble perception, Autism, Summary statistics, Facial expressions, Emotions

## Abstract

Ensemble perception, the ability to assess automatically the summary of large amounts of information presented in visual scenes, is available early in typical development. This ability might be compromised in autistic children, who are thought to present limitations in maintaining summary statistics representations for the recent history of sensory input. Here we examined ensemble perception of facial emotional expressions in 35 autistic children, 30 age- and ability-matched typical children and 25 typical adults. Participants received three tasks: a) an ‘ensemble’ emotion discrimination task; b) a baseline (single-face) emotion discrimination task; and c) a facial expression identification task. Children performed worse than adults on all three tasks. Unexpectedly, autistic and typical children were, on average, indistinguishable in their precision and accuracy on all three tasks. Computational modelling suggested that, on average, autistic and typical children used ensemble-encoding strategies to a similar extent; but ensemble perception was related to non-verbal reasoning abilities in autistic but not in typical children. Eye-movement data also showed no group differences in the way children attended to the stimuli. Our combined findings suggest that the abilities of autistic and typical children for ensemble perception of emotions are comparable on average.

## 1 Introduction

Human perception will often seek the summary, the texture or the ‘gist’ of large amounts of information presented in visual scenes. Large amounts of similar objects, for example, some books on a shelf, or the buildings of a city may give rise to group precepts – the percept of a book collection or a city view. Properties of group percepts – whether a book collection is tidied up or not, whether a view belongs to an old or a contemporary city – seem to be accessible rapidly and effortlessly, and with little awareness of details differentiating individual elements.

This ability to assess automatically the summary or ‘gist’ of large amounts of information presented in visual scenes, often referred to as ensemble perception or ensemble encoding, is crucial for navigating an inherently complex world (Chong and Treisman, 2003, Chong and Treisman, 2005, Haberman and Whitney, 2009, Sweeny et al., 2013). Given the processing limitations of the brain, it is often efficient to sacrifice representations of individual elements in the interest of concise, summary representations, which become available as the brain rapidly encodes statistical regularities in notions of a ‘mean’ or a ‘texture’ (Haberman and Whitney, 2012, Whitney et al., 2013).

Ensemble perception has been demonstrated consistently for low-level visual attributes, including size, orientation, motion, speed, position and texture (Ariely, 2001, Chong and Treisman, 2003, Parkes et al., 2001). More recently, studies have also demonstrated ensemble perception in high-level vision. In Haberman and Whitney (2007)'s initial work on ensemble perception – and on which the current study was based, three adult observers viewed sets of morphs (computer-generated continuous variations of expressions of the same face) ranging from sad to happy. Observers were then asked to indicate whether a subsequent test face was happier or sadder than the average expression of the set, a task that required creating an internal representation of an *average* of facial expressions in the first set. The precision with which the three observers completed this task was remarkably good. In fact, two of the three observers were as precise in discriminating ensemble emotions as they were in identifying the emotions of single faces (in a control task). In another task, the same observers viewed sets of emotional morphs and were subsequently asked to indicate which of two new morphs was a member of the preceding set. All three observers were unable to perform above chance in this condition, suggesting that observers were unable to encode information about individual face emotions, despite being able to encode seemingly effortlessly information about average emotions. Subsequent work has shown these effects for a range of facial attributes (gender, ethnicity, identity, emotion, attractiveness; Haberman et al., 2009, Haberman and Whitney, 2007, Haberman and Whitney, 2009, Haberman and Whitney, 2010, Haberman and Whitney, 2011, Neumann et al., 2013).

Sweeny et al. (2014) have also shown that ensemble perception of size is also present, though not yet fully developed early in development, in 4–6 year-old children. In the primary condition of their child-friendly task, participants saw two trees, each containing eight differently sized oranges, and were asked to determine which tree had the largest oranges overall. A secondary condition (see Sweeny et al., 2014) included experimental manipulations that allowed for the empirical simulation of performance in the primary condition with no ensemble coding strategies available–that is, as if participants gave their response after comparing the sizes of a single, randomly-chosen orange from each tree. The difference in accuracy between the primary and secondary conditions provided an estimate of the extent to which participants benefited from the use of ensemble perception strategies, the ‘ensemble coding advantage’ (Sweeny et al., 2014). They found significant ensemble coding advantages in both young children and adults, although children presented smaller such advantages than adults. An ideal observer model, which was also used to predict the minimum number of items integrated in the primary condition, suggested that both children and adults did not necessarily derive ensemble codes from the entire set of items (N = 16), while children integrated fewer items than adults (4.24 vs. 7.18 items, correspondingly, across both trees), consistent with the smaller ensemble coding advantage they exhibited.

In the current study, we examined ensemble perception of emotions in autistic children and adolescents, and contrasted these with typical children, adolescents and adults. Autism is a highly heterogeneous neurodevelopmental condition known for difficulties in social interaction and communication. However, autism is also characterised by atypicalities in sensation and perception (DSM-5; American Psychiatric Association, 2013; see Simmons et al., 2009; for review). Many studies have focused on the processing of social stimuli and of faces in particular. This literature presents a confusing picture. While many studies have reported that autistic children present pervasive difficulties in emotion discrimination (see Uljarevic and Hamilton, 2012; for review), other studies have found such difficulties specifically for negative or more complex emotions (Jones et al., 2011) or no difficulties at all (Ozonoff et al., 1990, Tracy et al., 2011).

Prominent theories have suggested difficulties in social perception might be driven by fundamental problems in global processing (weak central coherence; Happé and Frith, 2006) or a local-processing bias that leads to strengths in the processing of simple stimuli and to weaknesses in the processing of more complex stimuli (Mottron et al., 2006). We have suggested that the unique perceptual experiences of individuals with autism might be accounted for by attenuated prior knowledge within a Bayesian computational model of perceptual inference (Pellicano and Burr, 2012). This hypothesis posits limitations in the abilities of individuals with autism to derive, maintain and/or use efficiently summary statistics representations for the recent history of sensory input. Such limitations lead to a processing style where sensory input is modulated to a lesser extent by norms derived from prior sensory experience.

Karaminis et al. (2016) have recently demonstrated this account formally, in the context of temporal reproduction, using a Bayesian computational model for central tendency (Cicchini et al., 2012), which suggested that the phenomenon reflects the integration of noisy temporal estimates with prior knowledge representations of a mean temporal stimulus. Karaminis et al. (2016) contrasted the predictions of this ideal-observer model with data from autistic and typical children completing a time interval reproduction task (measuring central tendency) and a temporal discrimination task (evaluating temporal resolution). The simulations suggested that central tendency in autistic children was much less than predicted by computational modelling, given their poor temporal resolution.

Pellicano and Burr's (2012) hypothesis has also received empirical support from studies showing diminished adaptation in the processing of face (e.g., Pellicano et al., 2007, Pellicano et al., 2013) and non-face stimuli (e.g., Turi et al., 2015, van Boxtel et al., 2016). Such findings appear to generalise to ensemble perception, i.e., summary statistics representations derived on a trial-by-trial basis from stimuli presented *simultaneously* and for brief time intervals. Rhodes et al. (2015) have developed a child-appropriate version of a paradigm for ensemble perception of face-identity (Neumann et al., 2013), which they administered to 9 autistic children and adolescents and 17 age- and ability-matched typical children. These authors found reduced recognition of averaged identity in autistic participants.

In the current study, we evaluated two predictions, based on Pellicano and Burr (2012), for the patterns of performance of autistic and typical children and adolescents (aged between 6 and 18 years; hereafter ‘children’) by developing a developmentally-appropriate version of Haberman and Whitney (2007)'s paradigm for ensemble perception of emotions.

**Fig. 1:**
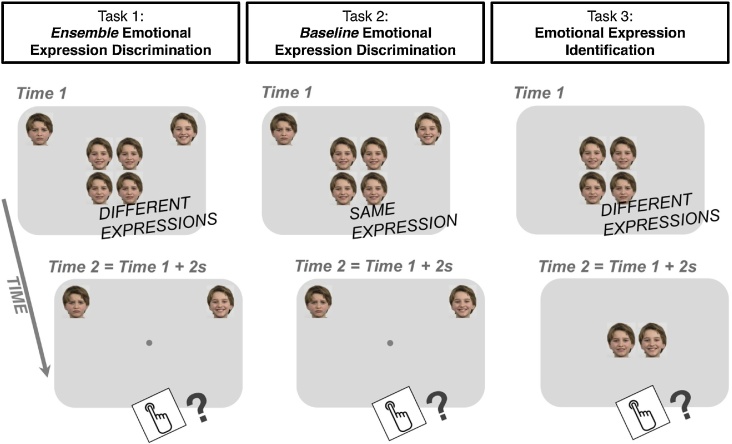
Paradigm structure. The paradigm comprised 1) an ensemble emotion discrimination task, 2) a baseline emotion discrimination task and 3) a face identification task. In Task 1, four *different* facial expressions (‘clones’) appeared near centre-screen for 2000 ms. Participants were instructed to indicate whether the four clones were *overall* more like the happy (upper right corner) or the sad clone (upper left corner). Task 2 (control) was identical to Task 1, apart from the fact that the four centre-screen emotional expressions were identical. In Task 3, four *different* facial expressions (‘clones’) appeared near centre-screen for 2000 ms and were followed by two more expressions. Participants were asked to indicate which of these two new faces was a member of the faces shown earlier. All tasks were child-appropriate versions of corresponding tasks in Haberman and Whitney (2007).

First, we predicted that autistic children should present difficulties in Task 1 assessing average emotion discrimination (see Fig. 1), evidenced by lower precision than typical children in the average relative to the baseline emotion discrimination task (as autistic children/adolescents might present general difficulties in emotion discrimination; Uljarevic and Hamilton, 2012). We further tested this prediction using computational modelling and eye-tracking methodologies. Computational simulations (akin to Sweeny et al., 2014) should suggest a weaker ensemble coding advantage and fewer items sampled in autistic children compared to typical children. Eye-tracking data could also reveal atypicalities in the ways autistic children attended to the stimuli (e.g., in the number of faces sampled).

Second, we predicted that autistic children should perform *better* than typical children in Task 3, identifying emotional morphs that had been previously presented to them. This advantage could be due to a greater reliance upon detailed representations of individual items, which are more important in this particular task, rather than on summary statistics (cf. Happé and Frith, 2006, Pellicano and Burr, 2012).

Finally, we also included a group of typical adults to examine developmental differences between children and adults in ensemble perception of emotions. We hypothesised that children were likely to show reduced abilities for ensemble perception compared to adults, similar to Sweeny et al.'s (2014) findings for the development of ensemble perception of size.

## 2 Material and methods

### 2.1 Participants

Participants' demographics are shown in Table 1. Thirty-five autistic children and adolescents (28 boys) aged between 7 and 16 years (M = 11.67; SD = 2.30) were recruited via schools in London and community contacts. All autistic children/adolescents had an independent clinical diagnosis of an autism spectrum disorder (ASD) and met the criteria for an ASD on the Autism Diagnostic Observation Schedule – 2 (ADOS-2) (Lord et al., 2012; cut-off score = 7) and/or the Social Communication Questionnaire – Lifetime (SCQ) (Rutter et al., 2003; cut-off score = 15) (see Table 1 for scores).

**Table 1:** Descriptive statistics for developmental variables for autistic and typical children.

Measures	Autistic	Typical	Statistical comparison
N	35	30	
Gender (n males: n females)	28: 7	19: 11	X^2^(2, N = 65) = 2.24, p = 0.13
Age (years)			
Mean (SD)	11.67 (2.30)	11.79 (3.18)	t(63) = 0.19
Range	7.42–16.83	6.99–17.60	p = 0.85
Verbal IQ^a^			
Mean (SD)	99.00 (19.07)	103.87 (9.29)	t(50.84) = 1.34,
Range	57–130	86–122	p = 0.19
Performance IQ^a^			
Mean (SD)	104.60 (16.32)	104.53 (15.78)	t(63) = 0.02,
Range	81–143	80–154	p = 0.99
Full-Scale IQ^a^			
Mean (SD)	101.80 (16.61)	104.60 (10.46)	t(58.13) = 0.82,
Range	73–136	88–132	p = 0.41
ADOS-2 score^b^			
Mean (SD)	10.33 (4.31)		
Range	2–20		
SCQ score^c^			
Mean (SD)	25.06 (8.31)	5.84 (4.19)	
Range	5–37	1–14	

*Notes*: ^a^Verbal, Performance and Full-Scale IQ were measured using the Wechsler Abbreviated Scales of Intelligence (WASI-II; Wechsler, 2011); ^b^ADOS-2: Autism Diagnostic Observation Schedule – 2 (cut-off score = 7; Lord et al., 2012), ^c^SCQ: Social Communication Questionnaire (score out of 40; Rutter et al., 2003). Higher scores on the ADOS-2 and the SCQ reflect greater autistic symptoms.

Thirty typically developing children and adolescents (19 boys), recruited from local London schools and community contacts, were matched with autistic children in terms of chronological age, t(63) = 0.19, p = 0.85, as well as on verbal IQ, t(50.84) = 1.34, p = 0.19, performance IQ, t(63) = 0.02, p = 0.99, and full-scale IQ, t(58.13) = 0.82, p = 0.41, measured by the Wechsler Abbreviated Scales of Intelligence – 2nd edition (WASI-II; Wechsler, 2011). All children were considered to be cognitively able (Full-scale IQ scores > = 70).

25 typical adults (11 men), aged between 18.70 and 44.40 years (M = 27.44; SD = 5.50) recruited from the University and community contacts, also took part.

Four additional autistic children and 4 typical children were tested but excluded from the analysis due to poorly fitting psychometric functions (R^2^ < 0.70, see below). Two additional autistic children were excluded because their IQ scores (WASI-II; Wechsler, 2011) were lower than 70.

### 2.2 Stimuli

Stimuli were two sets of 50 faces created by linearly interpolating two emotionally extreme faces, one with a sad expression and one with a happy expression of a boy (first set) and a girl (second set). The emotional extremes were chosen from the Radboud face database, based on their rankings of emotional intensity, clarity, genuineness and valence (Langner et al., 2010). Linear interpolation was performed using morphing software (FantaMorph, http://www.fantamorph.com), placing 250 landmarks in each endpoint face. The two sets of faces were taken to establish two continua of 50 emotional morphs, from sad to happy. Similar to Haberman and Whitney (2007), the distance between two successive morphs was one emotional unit, an arbitrary measure of the representation of happiness in successive morphs, assumed constant across the two continua and the same for the two sets. The saddest morphs were assigned an *emotional valence* value of 1, the happiest of 50, while the mean of the continua of 25.

Each face subtended 5.19° × 4.16° (h x w) of visual angle. Depending on the task, faces were presented in three possible configurations: i) in a passport photograph setup, i.e., as a group of four faces in a 2 × 2 grid, presented in the middle of the screen and over 10.81° x 8.79° of visual angle (including a 0.52° gap between individual faces); ii) as reference stimuli on the left and the right hand corner of the screen, 10.30° left or right and 5.19° above centre screen (this applied only to the saddest and the happiest morphs, respectively); iii) in a 1 × 2 (row x columns) grid, subtending 5.19° × 8.79° (see Procedure).

The experiments also included a centrally located fixation point, of grey colour and a diameter of 0.31° of visual angle.

Stimuli were presented on a light grey background (R = 227; G = 227; B = 227) of a 15.6-inch LCD monitor with 1920 × 1080 pixel resolution at a refresh rate of 60 Hz. All participants viewed the stimuli binocularly from a distance of 55 cm from the screen. We wrote the experiments in MatLab, using the Psychophysics Toolbox extensions (Brainard, 1997, Pelli, 1997, Kleiner et al., 2007).

### 2.3 Procedure

All child/adolescent and adult participants were given three tasks (see Fig. 1): 1) an ensemble emotion discrimination task; 2) a baseline emotion discrimination task; and 3) a facial expression identification task. The order of presentation of tasks was counterbalanced across participants, as was stimuli gender (i.e., for a given participant, all three tasks were based on a single set of faces).

Tasks were presented in the context of a child-friendly computer game, in which participants competed with characters from a popular animated movie (‘Despicable Me’) in activities involving judging emotions of *clones* of a boy or a girl or identifying clones who had been presented to them before.

#### 2.3.1 Task 1: ensemble emotion discrimination task

In this task, participants were told they would see a sad and a happy face appearing in the left and the right corner of the screen, correspondingly, and four *different* faces appearing near centre-screen for a limited time. They were instructed to indicate whether the four clones were *overall* more like the happy or the sad clone using the keyboard (keys ‘A’ and ‘L’). The experimenter used hand gestures to indicate the notion of ‘overall’.

As shown in Fig. 1 (left), each trial began with the reference stimuli presented near the two upper corners of the screen, along with the four faces in a 2 × 2 grid, presented in centre-screen for 2 s. The reference stimuli remained on screen for the duration of the trial.

Faces in the grid were all different from each other, separated by a standard distance of 6 emotional units. This meant that the emotional mean of each set was 9 units higher than the saddest face and 9 units lower than the happiest face in the set.

The task comprised 6 practice trials and 80 test trials. Practice trials familiarized the participant with the procedure and tested the following emotional means: 10, 40, 15, 35, 40 and 10 (in this order). Feedback was given. Practice trials were repeated (after a random permutation) if the participant produced incorrect judgements in at least two trials (three autistic children, two typical children).

The 80 test trials comprised 5 repetitions of 16 values of tested emotional means: 10, 12, 14, …, 40. No feedback apart from general positive encouragement was given to participants.

#### 2.3.2 Task 2: baseline emotion discrimination (control) task

This task (Fig. 1, middle) was identical in procedure to the average emotion discrimination task, including 6 practice trials and 80 test trials. In this task, however, the four faces in the 2 × 2 grid were indistinguishable. This implied a zero variance in which the emotional valence of the four faces coincided with the tested mean. We used four identical faces rather than just one face to achieve similar levels of perceptual complexity across the two tasks.

Participants were told they would see a sad and a happy clone appearing in the left and the right corner of the screen, correspondingly, and four *identical* clones appearing near centre-screen for a limited time (2 s). Participants were instructed to indicate whether the four clones were more like the happy or the sad clone using the keyboard.

Practice trials tested the following emotional means: 10, 40, 15, 35, 40 and 10 (in this order) and included feedback. They were repeated (after a random permutation) in the case of incorrect judgements in at least two of these trials (for one autistic child). Test trials included 5 repetitions of 16 values of tested emotional means: 10, 12, 14, …, 40. No feedback was given.

#### 2.3.3 Task 3: facial expression identification task

In this task, participants were told they would see four faces (clones) appear on the screen and then disappear. Two more faces would then appear. Participants were instructed to indicate which of the two faces was present in the group of four faces by making a corresponding keypress (A: left; L: right).

As shown in Fig. 1 (right), trials began by presenting a 2 × 2 grid of four different faces in centre-screen. These faces differed by 6 emotional units, i.e., similarly to the average emotion discrimination task. The emotional mean of the faces in the grid ranged from 10 to 40 with an increment of 2 emotional units (16 means X 5 trials = 80 trials in total).

After 2 s, the first set of faces disappeared and a new set of two faces (in a 1 × 2 grid) was shown in centre-screen. A target face in the second set was also a member of the first set of faces while the other was a distractor. The distance between the two faces in the second set could take one of three values: 3, 15 or 17 emotional units (that is, in each trial the distractor was 3, 15 or 17 units happier or sadder than the target face: Haberman and Whitney, 2007).

There were 6 demonstration trials and 80 test trials. Demonstration trials used target-distractor distances of 20 and 15 in this order: 20, –20, +15, –15, –20, +20, combined with the following emotional means values: 10, 40, 35, 15, 10, 40. These were repeated for five autistic children and four typical children.

Of the 80 test trials, 26 tested a target-distractor distance of 3 emotional units, 27 tested a distance of 15 emotional units, and 27 tested a distance of 17 units. Similar to the other two tasks, test trials considered 5 repetitions of 16 emotional means (10, 12, …, 40), assigned randomly to testing trials.

#### 2.3.4 General procedure

Children were tested individually in a quiet room at the University, at school or at home, and adults were tested in a quiet room at the University or at home. Testing lasted around 30–40 min. We collected eye-tracking data using a Tobii-X30 eye tracker, with a five-point calibration procedure repeated prior to each task. The WASI-II and the ADOS-2 were administered in later sessions.

The University's Faculty Research Ethics Committee approved this study. Adults gave their informed written consent and parents gave their consent for their child's participation prior to taking part.

### 2.4 Measurements and analysis

For ensemble and baseline emotional discrimination (Tasks 1 and 2), we fitted individual data from participants with bootstrapping (Efron and Tibshirani, 1993) with 200 repetitions and a ‘maximum likelihood’ fitting method (Watson, 1979). From the fitted curves we derived precision thresholds for each condition (the standard deviations of the fitted Gaussians). We conducted a mixed-design ANOVA on these measures with condition (ensemble and baseline emotion discrimination) as a repeated measures factor, and group (autistic, typical children, and adults) as a between-participants factor. For facial expression identification (Task 3), we measured accuracy in the three conditions of the tested distance (3, 15 and 17). We examined whether these measures differed from chance performance with two-tailed *t*-tests and examined differences across groups and conditions by conducting a 3 × 3 mixed-design ANOVA.

We also examined correlations between the so-obtained measures and age and performance IQ in the two groups of children, as well as correlations with measurements of autistic symptomatology in the group of autistic children, and correlations between precision thresholds in the two conditions in all groups. We calculated Pearson's linear correlations with permutation tests (100,000 permutations) and correcting for multiple comparisons using the “max statistic” method to adjust the p-values (Groppe et al., 2011). This method controlled for the family-wise error rate without being as conservative as Bonferroni correction (Groppe et al., 2011). We also used Fisher tests to assess whether correlations differed significantly between groups (for correlations that were significant in one group of children but not the other, N = 6), adjusting alpha levels for multiple comparisons with the Sidak method *(adjusted α = 1 – (1 – α)^1/N^, with α = 0.05).* Fisher tests were therefore conducted with adjusted alpha levels of 0.008 per test.

Participants' eye movements were analysed to provide additional insight into the way participants attended to the stimuli. We obtained usable eye-tracking data for 27 autistic children, 17 typical children and 14 adults. For these participants, we focused on trials where fixations were detectable at least 90% of the time, around 50% of the trials for all groups. We analysed recordings for these trials by deriving a scanning path for each participant and each trial. A scanning path was defined as a sequence of fixations in one of the four regions-of-interest, the square areas where the four facial expressions were shown on screen. A participant was taken to fixate in a given region if gaze remained in that region for more than 150 ms, otherwise these data were not included in the scanpath. For the scanning-path length and the mean number of samples scanned by a given participant in each task, we conducted mixed-design ANOVAs with task (ensemble, baseline emotion discrimination and facial identification) as a repeated measures factor, and group (autistic, typical children, and adults) as a between-participants factor.

### 2.5 Computational modelling

Our computational modeling aimed to assess the amount of information that participants used in the ensemble emotion discrimination task (rather than their mere performance in the same task). This is akin to the approach in Sweeny et al. (2014) on ensemble perception of size. Sweeny et al. (2014) included a control condition that allowed for the behavioural simulation of participants' abilities to perceive average size with no ensemble coding strategies available. Contrasting performance in this condition with performance in the principal condition, where participants were required to employ ensemble perception strategies, yielded the ensemble coding advantage. The ensemble coding advantage essentially measured the extent to which participants utilized ensemble perception strategies. Sweeny et al. (2014) also considered ensemble perception advantages predicted by ideal-observer models that assumed pooling of different amounts of items. They contrasted modelling results with human data to predict the number of individual items that participants integrated in the ensemble perception task in that study. Our computational modeling work aimed to perform a similar analysis and provide two measures characterizing the performance of individual participants: 1) the ensemble coding advantage, and 2) the number of samples that best accounted for the participant's actual performance in ensemble emotion discrimination.

Furthermore, our computational modeling aimed to contrast the performance of different groups in ensemble emotion discrimination (Task 1) given their baseline emotion discrimination abilities (Task 2, control). This was akin to the modeling approach in Karaminis et al. (2016), who assessed the amount of central tendency in temporal interval reproduction in autistic and typical children and adults (measured with a time interval reproduction task), taking into account their temporal resolution abilities (measured with a time discrimination task). This study showed that the patterns of performance of autistic children in time interval reproduction/discrimination were closer to the predictions of a computational model employed attenuated (compared to typical children and adults) prior knowledge representations of a mean interval. Here, the modeling aimed to assess whether the patterns of performance of autistic children are suggestive of less reliance on ensemble coding or of the integration of fewer items.

To address these issues, we developed an ideal observer model that simulated the performance of each participant in the ensemble emotion discrimination task if s/he gave her/his responses after subsampling one, two, or three randomly chosen faces or after sampling all four faces. In all four conditions for the sample size (N = 1, 2, 3 or 4), the ideal observer model for a given participant considered the same test trials as those presented to the participant. The model assumed noisy perception of the emotionality of the sampled faces, and noise was constrained by the performance of the participant in baseline emotion discrimination. We performed 500 Monte Carlo repetitions for each test trial. On each repetition, the emotionality values of faces were replaced with noise-perturbed values drawn from normal distributions centered on the actual emotionality values of the faces and with standard deviations equal to the precision of the participant in baseline emotion discrimination. Arguably, the inclusion of additional integration noise in the model would result in noisier estimates of the ensemble emotion. In that sense, the model is optimal or upper-bound. Therefore the model with a sample size parameter of N yielded the following estimate for an ensemble emotion expression:

$$perceived\;ensemble\;facial\;expression=\frac{\sum_{i=1}^{N}perceived\;emotion\;of\;sampled\;expression\;i}{N}$$

The perceived ensemble facial expression was then categorised as happy if it was higher than the point of subjective equality (PSE) in the fitted psychometric curve for this participant in baseline emotion discrimination and as sad otherwise.

The ideal observer model assumed no noise in the integration process per se. The integration of the noise-perturbed emotionality values for faces in the sample was therefore perfect, implying that the simulated precision of participants in average emotion discrimination was a lower-bound estimate, corresponding to optimal performance.

We used the results from the model simulations in two ways. First, we used the precision of the ideal observer model with N = 1 to calculate an *ensemble encoding advantage* for each participant – a measure of the extent to which a given participant benefited from ensemble coding strategies. It was calculated as the difference between the precision of the participant in average emotion discrimination and that of the ideal observer model with N = 1, normalised by the precision of the participant in baseline emotion discrimination:

$$Ensemble\;Coding\;Advantage=\frac{Precision\;in\;Task\;1-Precision\;of\;Ideal\;Observer\;Model\;with\;N=1}{Precision\;in\;Task\;2}$$

The ensemble coding advantage essentially contrasted the precision of a given participant in average emotion discrimination with the precision that the same participant would exhibit in this task if s/he responded after randomly sampling a single face from the test sets (similar to the behavioural simulation in Sweeny et al., 2014).

Second, and in a complementary analysis, we used the simulated precision values in all four ideal observer models (N = 1, 2, 3, and 4) for a given participant to estimate the number of samples that best accounted for the participant's actual performance in average emotion discrimination. This was done by fitting an exponential curve to the precision values obtained from the ideal observer models with N = 1, 2, 3 and 4, and then identifying the value of N (non-integer) that corresponded to the precision of the participant in average emotion discrimination in that curve.

## 3 Results

### 3.1 Ensemble (Task 1) and baseline (Task 2) emotion discrimination

Individual data from participants were well fit by cumulative Gaussian functions (autistic group: R^2^ = 0. 89 ± 0.07; typical group: R^2^ = 0.89 ± 0.06; adults: R^2^ = 0.93 ± 0.03). A preliminary analysis showed no effect of gender on performance in any task so data were collapsed across stimulus gender.

**Fig. 2:**
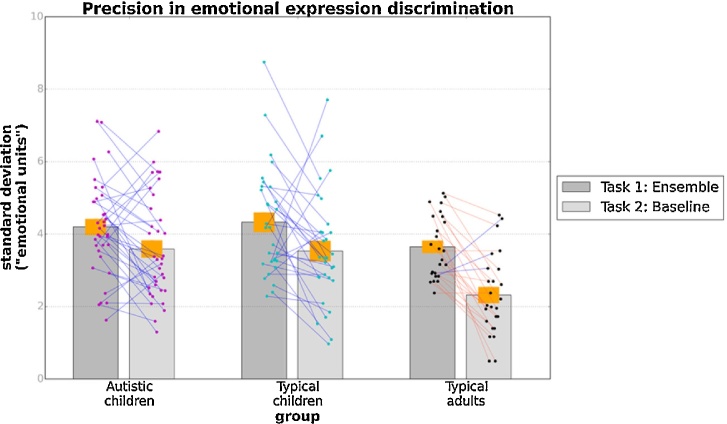
Mean precision for emotion discrimination (mean of standard deviations of the fitted psychometric curves) in the ensemble (Task 1) and individual emotion (Task 2) discrimination tasks for autistic children, typical children and typical adults. The orange bands correspond to ±1 SEM. Points superimposed on bars show individual variability, while blue lines connect data from the same participant.

First, we looked at participants' precision in ensemble and baseline emotion discrimination in autistic and typical children and adults. Fig. 2 shows precision thresholds, given by the standard deviation of the fitted cumulative Gaussian functions, for the three groups in the average and baseline emotion discrimination tasks. We conducted a mixed-design ANOVA with condition (ensemble and baseline emotion discrimination) as a repeated measures factor, and group (autistic, typical children, and adults) as a between-participants factor. There were significant effects of condition, F(1, 87) = 32.55, p < 0.001, n_p_^2^ = 0.27, and group, F(2, 87) = 6.28, p = 0.003, n_p_^2^ = 0.13, but no condition x group interaction, F(2, 87) = 1.75, p = 0.18, n_p_^2^ = 0.04. The analysis therefore suggested that, unlike Haberman and Whitney (2007), precision in ensemble emotion discrimination was worse than precision in individual emotion discrimination. This pattern was identical across groups. Planned contrasts suggested significant differences in precision between adults and typical children, t(87) = 0.95, p < 0.001, consistent with Sweeny et al. (2014). Contrary to expectations, there were no significant differences in precision between autistic and typical children (p = 0.79).

**Fig. 3:**
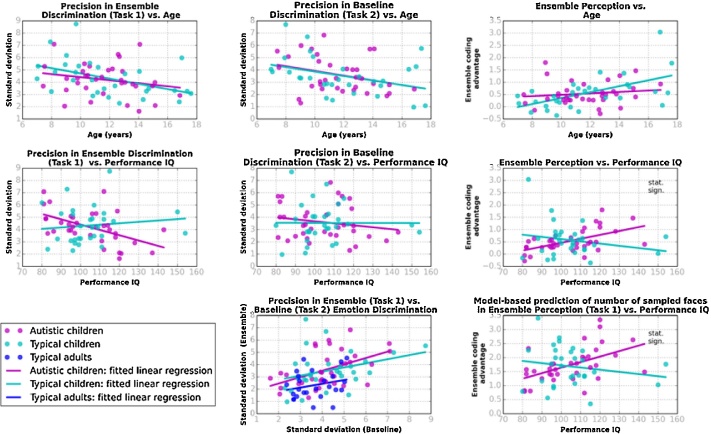
Within group individual variability. First row: Precision in ensemble emotion discrimination (Task 1), precision in baseline emotion discrimination (Task 2), and ensemble coding advantage plotted against chronological age. Second row: The same measures plotted against Performance IQ scores, obtained from the WASI-II (Wechsler, 2011). Third row: Precision in ensemble emotion discrimination (Task 1) plotted against precision in baseline emotion discrimination (Task 2) and model-based prediction for the number of samples plotted against Performance IQ scores. Dots correspond to individual data (cyan: typical children/adolescents; magenta: autistic children/adolescents; blue: adults). The continuous lines show fitted linear regressions. “Stat. sign.” indicates that the difference in the correlations between model-based measures and Performance IQ in autistic and typical children is statistically significant.

Next, we investigated within-group variability in ensemble emotion discrimination in autistic and typical children (Fig. 3). An examination of age-related improvements revealed no significant correlations between precision thresholds and ensemble emotion discrimination in typical [r(30) = –0.45, p = 0.11] and autistic children [r(35) = –0.21, p = 0.93]. However, autistic children's precision thresholds in ensemble emotion discrimination were highly correlated with their WASI-II Performance IQ scores [r(35) = –0.53, p = 0.01], a relationship not found in typical children [r(30) = –0.12, p = 1.00]. Fisher z-transformation tests suggested that the correlations between ensemble perception thresholds and age did not differ significantly in the two groups of children (z = 1.04, p = 0.30, two-tailed), while the correlations between the ensemble perception threshold and Performance IQ was not different in the two groups of children (z = 1.08, p = 0.07, two-tailed). No systematic relationships between precision thresholds in baseline emotion discrimination and chronological age or Performance IQ were found in either typical or autistic children (all *ps >* 0.28).

We also examined correlations between precision thresholds in ensemble and baseline emotion discrimination. These precision measures were strongly and positively correlated within the autistic group [r(35) = 0.48, p = 0.04], but not for typical children [r(30) = 0.33, p = 0.74] or adults [r(25) = 0.24, p = 0.25]. However, these correlations were not significantly different in autistic and typical children (z = 1.15, p = 0.25, two-tailed).

Finally, within the autistic group, there were no significant correlations between autistic symptomatology, as measured by the ADOS-2 and SCQ, and precision thresholds in baseline and ensemble emotion discrimination (all *ps >* 0.52).

### 3.2 Facial expression identification task (Task 3)

**Fig. 4:**
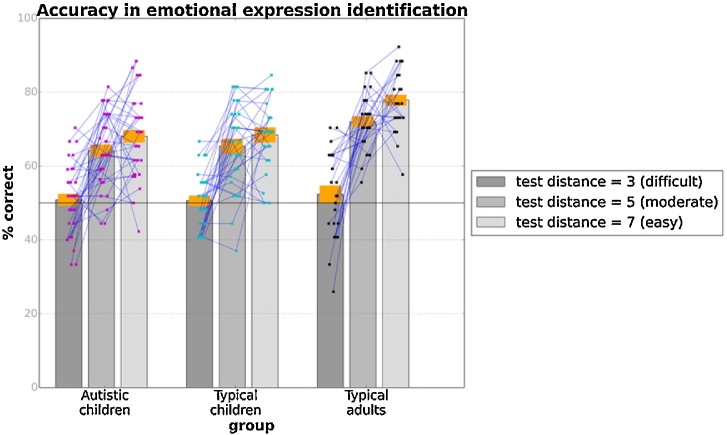
Mean accuracy in face identification task (Task 3) for three values of distance in emotional units between the two faces of the panel for autistic children, typical children and typical adults. Orange bands correspond to ±1 SEM. Points superimposed on bars show individual variability, while blue lines connect data from the same participant.

Similar to Haberman and Whitney (2007), we evaluated children and adults' accuracy in identifying morphs previously presented to them for the three conditions for the target-distractor emotional distance (3, 15 and 17). Accuracy rates for the three groups are shown in Fig. 4. As expected, and consistent with Haberman and Whitney (2007), accuracy was at chance for test stimuli with a target-distractor distance of 3 emotional units for all three groups [autistic: t(34) = 0.53, p = 0.60; typical: t(29) = 0.43, p = 0.67; adults: t(24) = 1.00, p = 0.33]. Unexpectedly, however, performance was above chance for test stimuli with distances of 15 or 17 (*ps <* 0.001).

We examined group differences in accuracy in the three conditions of the face-identification task by conducting a mixed-design ANOVA. There were significant effects of condition [Linear: F(1, 87) = 155.42, p < 0.001, n_p_^2^ = 0.64; Quadratic: F(1, 87) = 25.97, p < 0.001, n_p_^2^ = 0.23], group, F(2, 87) = 9.89, p < 0.001, n^2^ = 018, but no condition x group interaction [Linear: F(2, 87) = 2.51, p = 0.09, n^2^ = 0.06; Quadratic: F(1, 87) = 0.31, p = 0.74, n_p_^2^ = 0.01]. Planned comparisons suggested significant differences in accuracy between adults and typical children, t(74) = 0.06, p = 0.001, but, crucially, no differences were found between autistic and typical children (p = 0.99).

Examination of age-related improvements or improvements with Performance IQ revealed no significant correlations in emotional expression identification (all *ps >* 0.28). There were also no significant correlations between autistic symptomatology and accuracy in emotional expression identification (all *ps >* 0.86).

### 3.3 Computational modelling

**Fig. 5:**
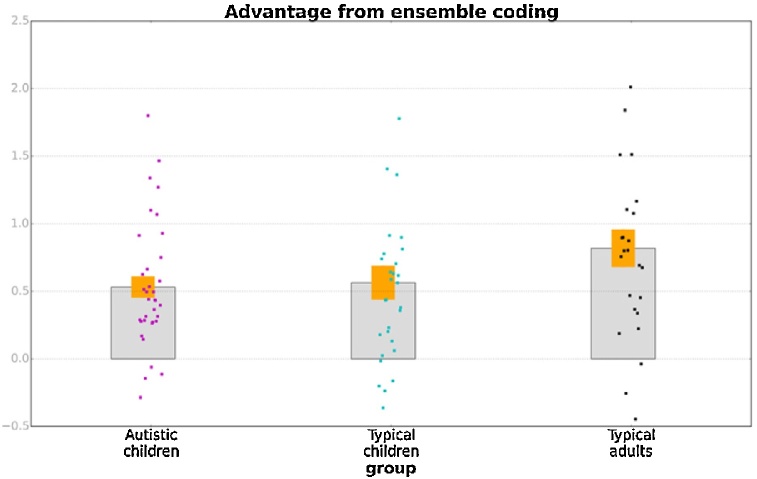
Ensemble coding advantage in the three groups of participants. Orange bands correspond to ±1 SEM, while points superimposed on bars show individual variability.

Fig. 5 shows the calculated ensemble coding advantages for the three groups [autistic: M = 0.53, SD = 0.47; typical: M = 0.56, SD = 0.68; adults: M = 0.81, SD = 0.70]. Ensemble perception advantages were significant for all three groups [autistic: t(34) = 6.74, p < 0.001; typical: t(29) = 4.52, p < 0.001; adults: t(24) = 5.98, p < 0.001]. Unexpectedly, there was no main effect of group, F(2, 87) = 1.79, p = 0.17. Planned contrasts suggested that adults did not present a greater ensemble coding advantage compared with typical children, t(87) = 1.50, p = 0.13, and, importantly, there was no significant difference between the two groups of children, t(87) = 0.13, p = 0.90.

**Fig. 6:**
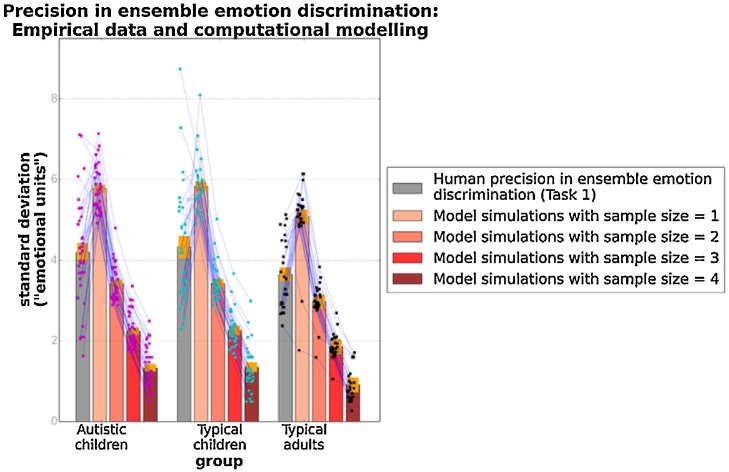
Precision in ensemble emotion discrimination: Empirical data and computational modelling. Orange bands correspond to ±1 SEM. Points superimposed on bars show individual variability, while blue lines connect empirical and simulation data for a given participant.

**Fig. 7:**
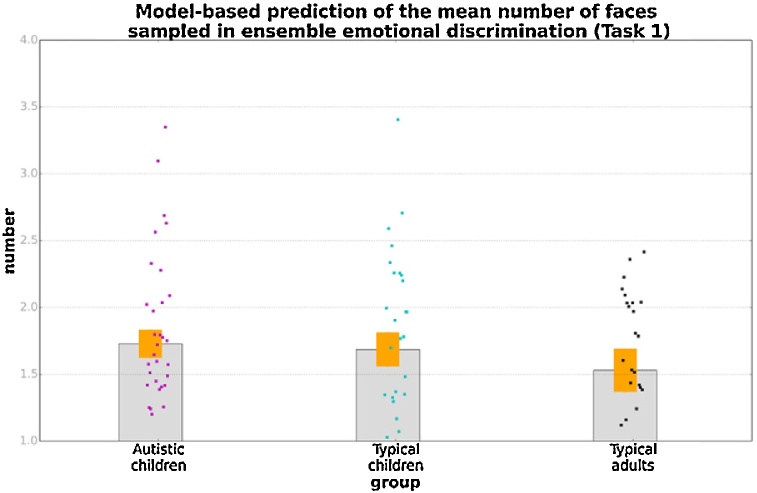
Predicted number of samples for the three groups of participants in the ensemble emotion discrimination task. Orange bands correspond to ±1 SEM, while points superimposed on bars show individual variability.

Fig. 6 presents precision in average emotion discrimination of the three groups (grey bars) along with the simulated precision obtained from the ideal observer models with N = 1, 2, 3, 4 (blue bars). The red lines connect model-predicted precision based on the data of individual participants. Fitting of an exponential curve (not shown in graph) to the model data yielded a non-integer N value representing the mean number of different emotional expressions sampled by a given participant in the average emotion discrimination task, according to the ideal observer model. Fig. 7 shows this measurement for the three groups. These were all significantly greater than 1 [autistic: t(34) = 6.93, p < 0.001; typical: t(25) = 5.40, p < 0.001; adults: t(24) = 3.54, p = 0.002]. A one-way ANOVA revealed no significant effect of group, F(2, 88) = 0.65, p = 0.52, suggesting that the model predicted no difference between the three groups in terms of the faces sampled in ensemble emotion discrimination.

Thus, the two model-based measures of ensemble perception did not present between-group differences as those found for precision in average emotion discrimination. However, the two model-based measures presented different patterns of within-group individual variability in autistic and typical participants, which were, importantly, largely consistent with patterns found in the empirical data for precision in average emotion discrimination (see Fig. 3). Ensemble coding advantage was highly correlated with age in typical children [r(30) = 0.56, p = 0.02], but not in autistic children [r(35) = 0.21, p = 1.00], though such a contrast was not present for the number of sampled faces [typical: r(30) = 0.32, p = 0.75; autistic: r(35) = 0.14, p = 1.00]. The two model-based measures were also highly correlated with Performance IQ within the autistic group [ensemble coding advantage: r(35) = 0.56, p = 0.01, mean number of sampled faces: r(36) = 0.52, p = 0.02], but not in the typical group [ensemble coding advantage: r(30) = –0.21, p = 0.87, mean number of sampled faces: r(36) = –0.18, p = 0.89]. Although Fisher tests suggested that there was no difference in the correlations between chronological age and ensemble coding advantage in the two groups of children (z = 1.61; p = 0.11, two-tailed), importantly, they showed that the correlations between Performance IQ and the two modelled based measures were significantly different between the groups (ensemble coding advantage: z = 3.24, p = 0.001, two-tailed; number of samples: z = 2.9, p = 0.004, two-tailed; adjusted α = 0.008). These correlations are shown in Fig. 3 (rightmost column, middle and lower plots). The between-group difference in the correlations between Performance IQ and ensemble coding advantage retained its significance within the adjusted alpha level when the outlying ensemble coding advantage of a typical participant was trimmed to 2 SD from the mean, (z = 2.82, p = 0.005, two-tailed).

Finally, autistic symptomatology did not correlate significantly with the model-based measures of ensemble perception [*ps* ≥ 0.97].

### 3.4 Eye-movement variables

**Fig. 8:**
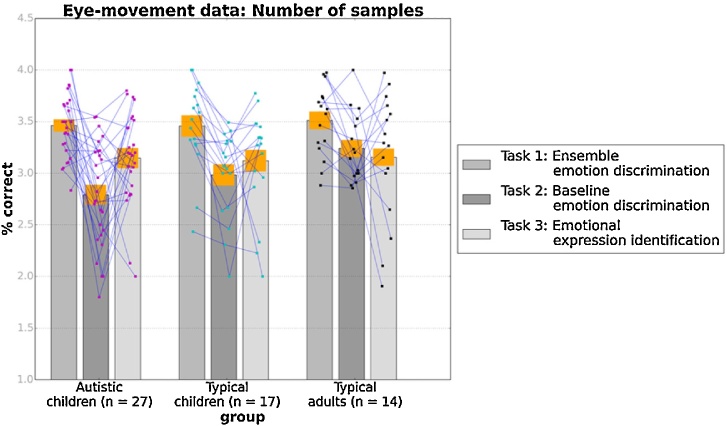
Mean number of different faces scanned by the participants in the three groups across test trials for the three tasks separately. Orange bands correspond to ±1 SEM, while blue lines connect data from the same participant.

Fig. 8 demonstrates the average number of different faces (morphs presented in different regions of interest) that the participants looked at in trials of the three tasks, for the three groups. A mixed-design ANOVA showed a significant quadratic effect of task on the number of faces sampled, F(1, 57) = 51.17, p < 0.001, n_p_^2^ = 0.4, but no significant effect of group, F (2, 57) = 1.81, p = 0.17, n_p_^2^ = 0.06, and no significant interaction between group and task [Linear: F(2, 57) = 2.17, p = 0.12, n_p_^2^ = 0.07; Quadratic: F(2, 57) = 0.91, p = 0.41, n_p_^2^ = 0.03]. Therefore, the three groups were indistinguishable in terms of the number of different morphs they sampled across the trials of the three tasks. They also presented a common pattern in which the number of different faces sampled was slightly higher in the average emotion discrimination than in the baseline emotion discrimination and the face-identification task.

Finally, we examined individual variability within the two groups of children with respect to eye-tracking variables. This analysis showed no systematic relationships between the way autistic or typical children attended to the stimuli and age or Performance-IQ and no significant correlations with autistic symptomatology in the autistic group (all *ps >* 0.65).

## 4 Discussion

A large body of empirical research has demonstrated the abilities of human perception to rapidly and automatically extract the summary or the gist of large amounts of information presented in visual scenes, also referred to as ensemble perception. We hypothesised that this fundamental ability for ensemble perception might be compromised in autistic children, who are held to present limitations in forming, accessing and/or using efficiently summary statistics representations for the recent history of their sensory input (Pellicano and Burr, 2012). Our hypothesis yielded two testable predictions: that (1) autistic children should present worse precision than typical children in a task involving ensemble perception of emotional morphed faces; and (2) autistic children might be more accurate than typical children in tasks that involve identification of individual faces (rather than encoding a summary emotion).

In direct contrast, we found no differences between autistic and typical children in terms of their precision in ensemble and baseline emotion discrimination, and in their accuracy in face identification. Our results showed that, relative to typical children, autistic children presented neither a limitation in ensemble perception nor an advantage in face identification. The two groups also did not differ in ensemble coding advantage and the number of samples integrated in each task, as suggested by the computational model. Eye-movement data further corroborated these findings: autistic and typical children looked at the same number of faces per trial on each task. Our analysis therefore showed that, on average, autistic and typical children performed largely similarly on our paradigm.

To examine further performance in ensemble emotional expression discrimination in isolation from baseline emotion discrimination, our study used computational modelling. Computational modelling suggested significant ensemble coding advantages for all three groups and that all groups integrated more than one face to determine the average emotion of a set. However, the three groups did not differ in these model-based measures. It is important to note that our modelling approach was conservative and the estimates of the participants' ensemble coding advantages and the number of faces they integrated in average emotion discrimination was lower-bound. While the model simulated baseline emotion discrimination taking into account estimates of noise (due to attention, motivation or decision-making) derived from the baseline emotion discrimination task, it did not include any late-stage noise in the integration process, like in other ideal-observer simulations of ensemble coding (Myczek and Simons, 2008). This late-stage noise would arguably increase the estimates of the precision of integration: that is, the model would predict higher levels of ensemble perception for a given value of precision in the average emotional expression discrimination task. In the absence of relevant empirical data, especially for differences between autistic and typical children, we opted to include no arbitrary constraints for late-stage noise in our model.

Eye-movement data on the other hand provided an upper-bound estimate of the number of faces that each participant integrated when completing each task (for example, looking at a face could not necessarily imply its integration with other test faces). Our eye-movement data did not suggest that differences in the way the three groups attended to the stimuli, in particular in the number of different faces scanned across trials.

We also investigated within-group individual variability in ensemble perception. This analysis revealed an interesting difference in the development of ensemble perception in autistic and typical children. In the group of autistic children, ensemble perception was closely related to their non-verbal reasoning ability. This relationship was not present in the group of typical children. This finding was supported by the computational modelling results rather than the empirical results in ensemble perception (Task 1). Computational modelling assessed performance in ensemble emotion discrimination (Task 1) focusing on the amount of information integrated by participants and ruling out differences in baseline emotion discrimination. Our results therefore suggest that ensemble perception *per se* presents an asymmetric relationship with general perceptual and reasoning abilities in autistic and typical children.

Indeed, our findings raise the possibility that ensemble perception might be fundamentally different in autistic and typical children. Ensemble coding in autistic children could be achieved through alternative cognitive strategies, possibly involving some kind of perceptual reasoning over individual emotional expressions. By contrast, in typical children, ensemble perception might involve domain-specific cognitive mechanisms.

We also showed that typical (and autistic) children performed worse than adults in all three tasks, presenting worse precision in baseline and average emotion discrimination and worse accuracy in the face-identification task. Our data suggested that abilities for ensemble perception of emotion, as well as the abilities for baseline emotion discrimination and emotional expression identification, are available early in development. These findings are consistent with the findings of Sweeny et al. (2014) on ensemble perception of a non-social stimulus, namely size, in younger children. However, our data could not demonstrate developmental improvements as correlations between precision measures in Tasks 1 and 2 or model-based measured of ensemble perception were not significant. Arguably, this might reflect a power issue. Eye-movement data too, showed no systematic correlations with age or performance IQ. Thus, differences in performance between children and adults in the three tasks, as well as individual variability in performance within the two groups of children, were not related to looking differences.

Our findings that ensemble perception of emotional expression is, on average, similar in autistic and typical children contrasts with those of Rhodes et al. (2015), who reported ensemble coding limitations in autistic individuals for face identity. One possibility is that this discrepancy is due to different mechanisms underlying the extraction of summary statistics for facial identity and emotions, consistent with theoretical proposals for the involvement of different pathways in the processing of invariant aspects of faces, such as identity, and changeable aspects, such as expression (Haxby et al., 2000; see also Calder and Young, 2005). However, we would also argue that the findings of Rhodes et al. (2015) warrant replication, especially since the sample of autistic individuals was very small (n = 9) and could not provide enough statistical power for the consideration of within-group variability.

Two patterns in our results, which characterized the performance of all three groups, were inconsistent with the original study by Haberman and Whitney (2007). First, we found that precision in ensemble emotion discrimination was worse than precision in baseline emotion discrimination. Haberman and Whitney (2007) found no difference between these two conditions for two of their three participants. Second, we found that accuracy in face identification was at above-chance levels for target-distractor emotional distances of 15 and 17. Haberman and Whitney (2007) had found that accuracy was at chance for all conditions of their face identification task. These discrepancies between our findings and Haberman and Whitney (2007) are likely to reflect a number of methodological differences (e.g., number of trials, number of participants, stimuli, use of reference stimuli on screen), which were introduced in our study to develop a child-appropriate version of the original paradigm. Our findings that accuracy in face identification was at above-chance levels (for some conditions) suggested that children, adolescents and adults present abilities for ensemble perception, as well as abilities to represent individual items. This pattern is consistent with other studies on ensemble perception (Kramer et al., 2015, Neumann et al., 2013).

Our results also suggested that autistic children/adolescents had no problems in emotion perception, either in the baseline or the ensemble discrimination tasks or even in the identification of facial expressions. This finding is in line previous studies reporting no differences between autistic and typical children in emotion discrimination and identification tasks (Ozonoff et al., 1990, Tracy et al., 2011).

It is possible that our task was simply not sufficiently difficult to detect differences between autistic and typical children in any of the three tasks. However, this is unlikely, given the significant age-related differences between children and adults. Another potential limitation is that our results reflect sampling issues and that ensemble processing abilities would be not as robust in a group of autistic children with poorer baseline emotion discrimination abilities (as suggested by the correlations between performance in the two tasks in our data).

Nevertheless, the different individual variability profiles of the two groups of children in ensemble perception demonstrate that it is important for future studies on ensemble perception to consider individual differences. Our results also demonstrate the need to refine prominent theories of autistic perception, for example theories suggesting limitations in global processing (Happé and Frith, 2006), the processing of more complex stimuli (Mottron et al., 2006) and, of course, the hypothesis of attenuated prior knowledge (Pellicano and Burr, 2012). To account for our data, these theories need to accommodate mechanistic accounts for how qualitatively different strategies might give rise to similar overall performance in ensemble perception in typical development and the autism spectrum.

Gaining knowledge of the temporal dynamics of ensemble perception would be a valuable way to address this issue. For example, our results suggest that ensemble perception could be less rapid as a process in autistic children, due to its greater reliance on some kind of perceptual reasoning. Our study, and the original study of Haberman and Whitney (2007), obtained responses after the stimuli have remained on screen for 2s, and therefore could not provide reliable measures of reaction times. Studies with time-contingent designs, more demanding stimuli, as well as electrophysiological approaches could be used to assess the rapidity of ensemble perception in typical development and autism.

Theories of autistic perception and ensemble perception also need to consider the possibility of efficient compensation for ensemble perception in autism. Developmental and other studies on ensemble perception have argued that its early emergence and ubiquity reflect its fundamental importance in perception and, in the case of social stimuli, in the development of social behaviour and cognition (Haberman and Whitney, 2012, Sweeny et al., 2014, Neumann et al., 2013, Rhodes et al., 2015, Whitney et al., 2013). A number of previous studies have also established that autistic individuals present atypical adaptation to various dimensions of facial stimuli (e.g. Pellicano et al., 2007, Pellicano et al., 2013), suggestive of limitations in their abilities to extract norms for faces seen during the recent history of sensory input. Such limitations might give rise to difficulties in ensemble perception, with profound effects in their ability to adapt and respond to social environments. It is possible that these difficulties are compensated in autism through the use of domain-general perceptual reasoning over individually perceived stimuli. If this is the case, adults on the autism spectrum should also show a reliance of abilities for ensemble perception on perceptual reasoning abilities.

Finally, it is important to ask whether our findings are specific to ensemble perception of facial attributes or whether they generalise to low-level stimuli (Sweeny et al., 2014). An interesting possibility is that qualitative differences in ensemble perception should manifest in domains where autistic individuals present diminished perceptual adaptation (e.g., numerosity: Turi et al., 2015; audiovisual adaptation: Turi et al., 2016), rather than domains where adaptation is similar to typical development (e.g., perceptual causality: Karaminis et al., 2015).

## Conflict of Interest

None.
